# *Brucea javanica* oil emulsion injection (BJOEI) as an adjunctive therapy for patients with advanced colorectal carcinoma: A protocol for a systematic review and meta-analysis

**DOI:** 10.1097/MD.0000000000021155

**Published:** 2020-07-02

**Authors:** Chunhong Xu, Xinxin Guo, Changhui Zhou, Hualing Zhang

**Affiliations:** a Department of Gastroenterology; b Department of Central Laboratory, Liaocheng People's Hospital, Liaocheng, Shandong Province, China.

**Keywords:** *Brucea javanica* oil emulsion injection, colorectal cancer, efficacy, meta–analysis, safety

## Abstract

**Background:**

*Brucea javanica* oil emulsion injection (BJOEI) has been widely applied as a promising adjunctive drug for colorectal carcinoma (CRC). However, the exact effects and safety of BJOEI remains controversial. In this study, we aimed to summarize the efficacy and safety of BJOEI for the treatment of advanced CRC through the meta-analysis, in order to provide scientific reference for the design of future clinical trials.

**Methods:**

Eligible prospective controlled clinical trials were searched from PubMed, Cochrane Library, Google Scholar, Medline, Web of Science (WOS), Excerpt Medica Database (Embase), Chinese BioMedical Database (CBM), China Scientific Journal Database (VIP), China National Knowledge Infrastructure (CNKI) and Wanfang Database. Papers in English or Chinese published from January 2000 to May 2020 will be included without any restrictions. The clinical outcomes including therapeutic effects, quality of life (QoL), immune function and adverse events, were systematically evaluated.

Study selection and data extraction will be performed independently by 2 reviewers. Review Manager 5.3 and Stata 14.0 were used for data analysis, and a fixed or random-effect model will be used depending upon the heterogeneity observed between trials. Subgroup and meta-regression analysis will be carried out depending on the availability of sufficient data.

**Results:**

The results of this systematic review will be published in a peer-reviewed journal.

**Conclusion:**

Our study will draw an objective conclusion of the effects and safety of BJOEI for advanced CRC, and provide a helpful evidence for clinicians to formulate the best postoperative adjuvant treatment strategy for CRC patients.

INPLASY registration number: INPLASY202060014.

## 1 Introduction

Globally, colorectal cancer (CRC) is the third most commonly diagnosed malignancy and the second most frequent cause of cancer-related death.^[1,2]^ In recent years, the incidence of CRC has significantly raised with about 1.8 million new cases every year, and caused 861,700 deaths worldwide in 2018.^[1,2]^ Besides an ageing population, genetic factors and dietary habits, unfavorable risk factors such as obesity, work pressure, lack of physical exercise and smoking increase the risk of CRC.^[3–5]^ CRC is also one of the worst gastrointestinal malignancies, with a strong tendency of invasion and metastasis. Despite the improvement of diagnostic and therapeutic methods in the past decades, the prognosis of CRC remains unsatisfactory.^[3,6,7]^ More than half CRC patients already have advanced or metastatic lesions when diagnosed, due to the lack of noticeable clinical symptoms at early stage, and the 5-year survival rate of advanced CRC patients was only 13.1%.^[8,9]^ Currently, the clinical treatment of CRC mainly includes chemotherapy, radiotherapy, surgical resection alone or combined strategy.^[5,6]^ However, their applications are limited by failing to thoroughly eliminate tumor cells, drug resistance and other adverse effects.^[6]^ Therefore, more effective and safer treatments were urgently required.

As complementary and alternative medicine, traditional Chinese medicine has become one of an effective assistant method for cancer comprehensive treatment.^[10–15]^ More and more researchers indicated that the combination of Chinese and Western medicine for CRC may be the potential trend of clinical treatment development in future.^[10,16,17]^ As one of the famous traditional Chinese herbal medicine preparations, *Brucea javanica* oil emulsion injection (BJOEI, also named yadanzi oil in China) was takes Brucea Jen petroleum ether extracts as raw material and purified soybean lecithin as emulsifier,^[18–23]^ and is often employed as adjunctive therapy in combination with radiochemotherapy for malignancies including CRC.^[18–23]^ BJOEI contains oleic acid, linoleic acid, palmitic acid, arachidonic acid, stearic acid, and other anticancer active ingredients,^[16]^ which mainly produced in the People's Republic of China's coastal tropical and subtropical regions such as Hainan, Guangxi, Guangdong, Yunnan, and other places.^[19,20]^

In 1985, phase III clinical trials were completed and BJOEI was officially launched in China after final approval from the Ministry of Health of the People's Republic of China.^[20]^ Previous studies suggest that the anti-tumor mechanisms of BJOEI might be attributed to the following aspects:

(1) BJOEI can significantly inhibit the proliferation of cancer cells by inhibiting the deoxyribonucleic acid (DNA) synthesis and arresting the tumor cell division cycle at G2/M.^[19–21,23]^
(2) BJOEI can induces cancer cell apoptosis through activation of caspase apoptotic pathway by upregulation of the expression of caspase-3 and caspase-9 proteins and inhibition of the expression of nuclear factor kappa B, phosphoinositide 3-kinases and protein Kinase B.^[19–21]^
(3) It also can exert the anti-tumor efficiency through disrupting the cellular energy metabolism, depressing the expression of vascular endothelial growth factor, and inhibiting the migration and invasion of tumor cells.^[21]^

Other studies suggest that BJOEI can effectively reverse the multidrug resistance (MDR) of tumor cells and increase the sensitivity of cancer cells to chemotherapeutic agents.^[20,22]^

Many studies have proved that BJOEI can perform a synergetic antitumor effect by improving tumor response, improving the quality of life (QoL) and reducing the incidence of adverse events during radiochemotherapy.^[24–27]^ Despite the intensive clinical studies, its clinical efficacy for advanced CRC was still not systematically evaluated. We are prepared to summarize the efficacy and safety of BJOEI treatment for CRC at advanced stages through the meta-analysis, in order to provide scientific reference for the design of future clinical trials.

## 2 Study aim/Objective

The aim of our study is to propose a protocol for a systematic review and meta-analysis to systematically evaluate the efficacy and safety of BJOEI adjuvant therapy combined with conventional treatment for advanced CRC.

## 3 Methods

The protocol of systematic review and meta-analysis will be reported according to the Preferred Reporting Items for Systematic Reviews and Meta-Analyses Protocols (PRISMA-P) guidelines.^[28]^ Our protocol has been registered on the International Platform of Registered Systematic Review and Meta-Analysis Protocols (INPLASY). The registration number was INPLASY202060014 (DOI number is 10.37766/inplasy2020.6.0014, https://inplasy.com/inplasy-2020-6-0014/). This meta-analysis is a secondary research which based on some previously published data. Therefore, the ethical approval or informed consent was not required in this study.

### 3.1 Search strategy

To perform a comprehensive and focused search, experienced systematic review researchers will be invited to develop a search strategy. The plan searched terms are as follows: “colon cancer” or “colon neoplasm” or “colon carcinoma” or “colon tumor” or “rectal cancer” or “rectal neoplasm” or “rectal carcinoma” or “rectal tumor” or “colorectal cancer” or “colorectal neoplasm” or “colorectal carcinoma” or “colorectal tumor” or “CRC” or “CC” or “RC” and “Javanica oil emulsion injection” or “Brucea javanica oil emulsion” or “Brucea javanica oil emulsion injection” or “BJOEI” or “BJOE injection” or “Yadanzi” or “Yadanzi injection” or “Ya-dan-zi injection” et al. An example of search strategy for PubMed database shown in Table 1 will be modified and used for the other databases.

**Table 1:**
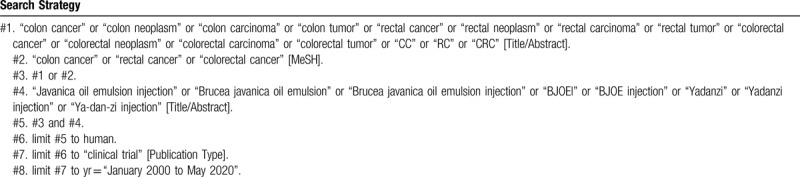
Searching strategy in PubMed.

### 3.2 Eligibility criteria

#### 3.2.1 Participant or population

Patients must be cytologically or pathologically confirmed as having CRC at a clinically advanced stage. There will be no restrictions regarding gender, age, region, racial, economic and education status. Patients with other malignancies or non-primary CRC are not included.

#### 3.2.2 Intervention

In the experimental group, advanced CRC patients must be treated with conventional treatment (such as chemotherapy, radiotherapy, and immunotherapy, et al) combined with BJOEI mediated therapy.

#### 3.2.3 Comparator

In the control group, CRC patient treated with the same conventional treatment as intervention group in the same original research.

#### 3.2.4 Studies designs to be included

All available randomized controlled trials (RCTs) and high-quality prospective cohort studies that investigated the efficacy and safety of BJOEI-mediated therapy in patients diagnosed with CRC will be included in this systematic review.

#### 3.2.5 Exclusion criteria

Articles without sufficient available data, non-comparative studies, non-peer reviewed articles, literature reviews, meta-analysis, case reports and series, meeting abstracts, animal studies, letter to the editor, editorials, commentaries, and other unrelated studies will be all excluded from analysis.

### 3.3 Information sources

Electronic databases including PubMed, Cochrane Library, Google Scholar, Medline, Web of Science (WOS), Excerpt Medica Database (Embase), Chinese BioMedical Database (CBM), China Scientific Journal Database (VIP), China National Knowledge Infrastructure (CNKI) and Wanfang Database, will be systematically searched for eligible studies from January 2000 to May 2020. Language is limited with English and Chinese.

### 3.4 Types of outcome measures

#### 3.4.1 Main outcomes

The primary outcomes in present analysis included short-term and long-term clinical efficacy, and adverse effects (AEs) according to Organization (WHO) criteria and Response Evaluation Criteria in Solid Tumors 1.1 (RECIST Criteria 1.1).^[29]^

(I) Short-term clinical efficacy: the short-term tumor response included complete response (CR), partial response (PR), stable disease (SD), progressive disease (PD), overall response rate (ORR) and disease control rate (DCR). ORR was defined as the sum of CR and PR, and DCR was the sum of CR, PR and SD.
(II) Long-term clinical efficacy: 1–5 year Overall survival (OS, which is defined as the time from the date of randomization to death from any cause); 1–5 year progression free survival (DFS, which is the time from date of random assignment to date of recurrence or death).
(III) Adverse events: toxicity was graded from 0 to IV in severity on the basis of the WHO recommendations.

#### 3.4.2 Additional outcomes

Secondary outcomes will include:

(I) QoL: QoL was evaluated using Karnofsky score;
(II) Immune function indicators: the immune function of CRC patients was assessed in terms of CD3^+^, CD4^+^, CD8^+^, NK cells percentage, and CD4+/CD8+ cell ratios.

### 3.5 Data collection and analysis

We will adopt the measures described in the Cochrane Handbook for Systematic Reviews of Interventions to pool the evidence.^[30]^

#### 3.5.1 Study selection and management

**Figure 1:**
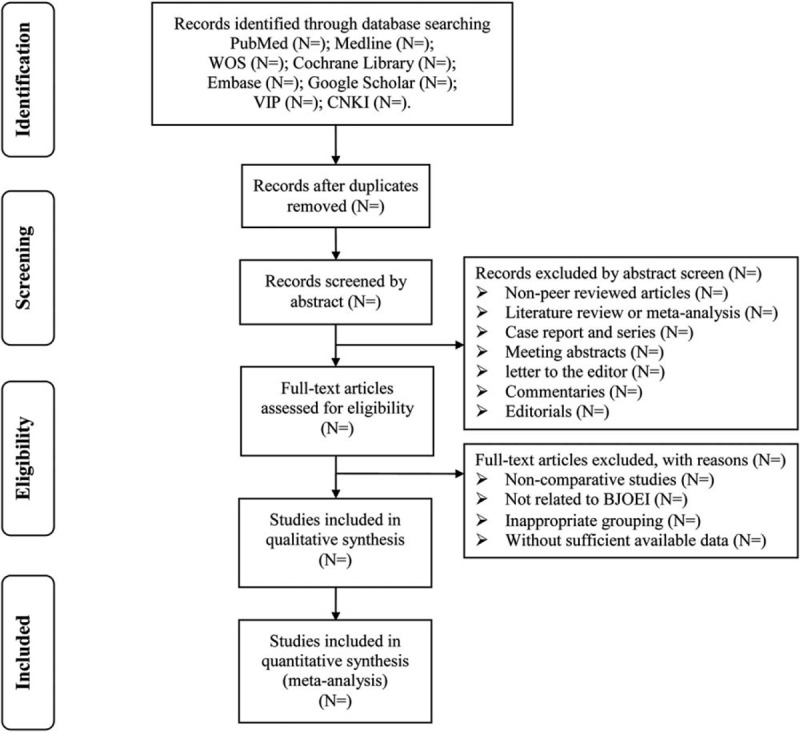
Study selection process for the meta-analysis. CBM = Chinese Biomedical Literature Database, CNKI = China National Knowledge Infrastructure, EMBASE = Excerpt Medica Database, VIP = China Scientific Journal Database, WOS = web of science.

Endnote X7 software will be used for literature managing and records searching. Two investigators (Xu CH and Guo XX) will be reviewed independently to identify potential trials by assessing the titles and abstracts and identify whether the trials meet the inclusion criteria. The full text will be further reviewed to exclude irrelevant studies or determine potential eligible studies. Disagreements between the two authors will be resolved by discussing with the third reviewer (Zhou CH). A PRISMA-compliant flow chart (Fig. 1) will be used to describe the selection process of eligible literatures.

#### 3.5.2 Data extraction and management

Two investigators (Xu CH and Guo XX) will be responsible for the data extraction independently according to the Cochrane Handbook for Systematic Reviews of Intervention.

The following data will be extracted from eligible literatures:

- Study characteristics: country of study, the first author, year of publication, sample size, periods of data collection, total duration of study and follow-up duration, et al.
- Participant characteristics: tumor stage (staging of the tumor according to the AJCC TNM classification for esophageal cancer), age, gender, ethnicity, pathology diagnosis, pathologic tumor size, inclusion and exclusion criteria, et al.
- Interventions: therapeutic means, manufacturer of the drugs, dosage of BJOEI, administration route and cycles and duration of treatment, et al.
- Outcome and other data: CR, PR, SD, PD, ORR, DCR, OS, DFS, QoL, immune indexes (CD3^+^, CD4^+^, CD8^+^, NK cells percentage, and CD4+/CD8+ cell ratios) and adverse effects, et al. For survival outcomes, Hazard ratios (HRs) with corresponding 95% confidence intervals (CIs) will be extracted from trials or be estimated from Kaplan–Meier survival curves by established methods.^[31]^

*Dealing with missing data:* we will attempt to contact the authors to request the missing or incomplete data. If those relevant data are not acquired, they will be excluded from the analysis. Any disagreements will be resolved by discussion, and a third reviewer (Zhou CH) will make the final decision. Excluded studies and the reasons for exclusion will be listed in a table.

### 3.6 Quality assessment/Risk of bias analysis

The quality of the included clinical trials will be assessed independently by 2 investigators (Xu CH and Guo XX) in terms of random sequence generation (selection bias), allocation concealment (selection bias), blinding of participants and personnel (performance bias), blinding of outcome assessment (detection bias), incomplete outcome data (attrition bias), selective outcome reporting (reporting bias) and other bias, according to the guidance of the Cochrane Handbook for Systematic Review of Interventions.^[30,32]^ Evidence quality will be classified as low risk, high risk, or unclear risk of bias in accordance with the criteria of the risk of bias judgment. EPOC guidelines will be used to assess the risks of non-RCTs.^[33]^ Any disagreements will be resolved via discussion with a third researcher (Zhou CH). If necessary, consulting with the fourth author (Zhang HL).

### 3.7 Strategy of data synthesis

Data from studies judged to be clinically homogeneous will be pooled using Review Manager 5.3 (Nordic Cochran Centre, Copenhagen, Denmark) and Stata 14.0 (Stata Corp., College Station, TX) statistical software. Heterogeneity between studies will be assessed using the Cochran's Q and Higgins *I*^*2*^ statistic. *P* < .1 for the Chi^2^ statistic or an *I*^*2*^ > 50% will be considered as showing considerable heterogeneity.^[34]^ A fixed effect model will be used to calculate the outcomes when statistical heterogeneity is absent; otherwise, the random effects model was considered according to the DerSimonian and Laird method.^[35]^ The Mantel–Haenszel method will be applied for pooling of dichotomous data and results will be presented as relative risk (RR) with their 95% confidence intervals (CIs). Inverse variance method will be used for pooling of continuous data and results will be presented as standardized mean difference (SMD) with their 95% CIs. A two-tailed *P* < .05 was considered statistically significant.

### 3.8 Subgroup and meta-regression analysis

If the data are available and sufficient, subgroup and meta-regression analysis will be conducted to explore the source of heterogeneity with respect to age, gender, region, tumor stage, course of treatment and therapeutic regimens.

### 3.9 Sensitivity analysis

Sensitivity analysis will be conducted to assess the reliability and robustness of the aggregation results via eliminating trials with high bias risk. A summary table will report the results of the sensitivity analyses.

### 3.10 Publication bias analysis

We will detect publication biases and poor methodological quality of small studies using funnel plots if 10 or more studies are included in the meta-analysis. Begg and Egger regression test will be utilized to detect the funnel plot asymmetry.^[36–38]^ If reporting bias is suspected, we will consult the study author to get more information. If publication bias existed, a trim-and-fill method should be applied to coordinate the estimates from unpublished studies, and the adjusted results were compared with the original pooled RR.^[39,40]^

### 3.11 Evidence evaluation

The evidence grade will be determined by using the guidelines of the Grading of Recommendations, Assessment, Development, and Evaluation (GRADE). The quality of all evidence will be evaluated as four levels (high, moderate, low, and very low).^[30,41,42]^

### 3.12 Dissemination plans

We will disseminate the results of this systematic review by publishing the manuscript in a peer-reviewed journal or presenting the findings at a relevant conference.

## 4 Discussion

CRC is a highly malignant tumor, and current treatment methods only have a modest survival benefit. Therefore, therapies that could significantly improve OS and have fewer side effects are what we need to pursue now. Currently, it has reported that medicinal herbs have a unique advantage in CRC therapy by inhibiting the growth of cancer cells, enhancing immunity, decreasing cancer relapses and metastases, mitigating the progress of the disease and improving the QoL of CRC patients.^[43–45]^ As an effective Chinese herbal medicine preparation, BJOEI has been widely used alone or combined with radiochemotherapy for the treatment of diverse malignant tumors.^[19–23]^

### 4.1 Strengths and limitations of this study

Even though there was statistical analysis of published clinical trials, the exact therapeutic effects of BJOEI mediated therapy for CRC were remains controversial. Thus, in-depth knowledge of the efficacy and safety of BJOEI is needed. This systematic review will provide a helpful evidence for clinicians to formulate the best postoperative adjuvant treatment strategy for patients with advanced CRC, and also provide scientific clues for researchers in this field.

Moreover, immune system reconstruction is one of the critical factors to effectively treat malignancies. T lymphocyte subsets (CD3^+^, CD4^+^, CD8^+^ cell subsets, and CD4^+^/CD8^+^ ratio) play an important roles in antitumor immunity.^[6,22,46]^ Studies have shown that patients with advanced cancer showed decreased immune function and natural killer cell activity, and exhibiting imbalance of T lymphocytes percentage.^[6,22,47,48]^ Chemotherapy agents could also have a negative impact on the immune function, therefore further affecting the therapeutic effect.^[22]^ In this study, we will summarize and analyze the immune indicators of CRC patients before and after treatment, in order to explore the therapeutic effect of BJOEI on patients from the aspect of immune function.

The systematic review will also have some limitations. There may be a language bias with the limitation of English and Chinese studies. In addition, Clinical heterogeneity may exist for different tumor stage and ages of CRC patients, dosage of BJOEI, and duration of treatment.

## Author contributions

Zhang HL and Xu CH conceived the concept and designed the study protocol. Zhang HL and Xu CH tested the feasibility of the study. Xu CH and Guo XX wrote the manuscript. Zhang HL, Zhou CH and Xu CH provided methodological advice, polished and revised the manuscript. All authors approved the final version of the manuscript.

**Conceptualization:** Chunhong Xu, Hualing Zhang.

**Funding acquisition:** Changhui Zhou.

**Investigation:** Chunhong Xu, Xinxin Guo, Changhui Zhou.

**Methodology:** Chunhong Xu, Xinxin Guo, Changhui Zhou.

**Project administration:** Hualing Zhang.

**Supervision:** Chunhong Xu, Hualing Zhang.

**Writing – original draft:** Chunhong Xu, Xinxin Guo.

**Writing – review & editing:** Chunhong Xu, Hualing Zhang.
